# Clozapine-induced obsessive-compulsive symptoms in schizophrenia: Clinical and cognitive determinants of dysfunctional checking

**DOI:** 10.1017/S0033291724003350

**Published:** 2025-03-18

**Authors:** Marjan Biria, Paula Banca, Isaac Jarratt Barnham, Aleya A. Marzuki, Nuria Segarra, Engin Keser, Akeem Sule, Marija Farrugia, Qiang Luo, Naomi Fineberg, Emilio Fernandez-Egea, Trevor W. Robbins

**Affiliations:** 1 Department of Psychology, University of Cambridge, Cambridge CB2 3EB, UK; 2 Behavioural and Clinical Neuroscience Institute, University of Cambridge, Cambridge CB2 3EB, UK; 3 Division of Psychiatry and Division of Psychology and Language Sciences, University College London, London, UK; 4 Cambridge Psychosis Centre, Cambridgeshire and Peterborough NHS Foundation Trust, Cambridge, UK; 5 Pembroke College, University of Oxford, St. Aldates, Oxford OX1 1DW, UK; 6 Department of Psychiatry and Psychotherapy, Medical School and University Hospital, Eberhard Karls University of Tübingen, Tübingen, Germany; 7 German Center for Mental Health (DZPG), Tübingen, Germany; 8 Cambridgeshire and Peterborough NHS Foundation Trust, Cambridge, UK; 9 Genetic and Developmental Psychiatry Centre, King’s College London, London, UK; 10 Department of Psychiatry, School of Clinical Medicine, University of Cambridge, Cambridge, UK; 11 Centre for Computational Psychiatry, Institute of Science and Technology for Brain-Inspired Intelligence, Fudan University, Shanghai 200433, China; 12 Hertfordshire Partnership University NHS Foundation Trust, National Health Service, University of Hertfordshire, Hatfield, UK; 13 Department of Psychiatry, University of Cambridge, Cambridge, UK

**Keywords:** checking, cognitive flexibility, OCD, schizophrenia, spatial working memory

## Abstract

**Background:**

Obsessive-compulsive symptoms (OCS) emerge in a significant proportion of clozapine-treated schizophrenia patients, affecting social functioning and increasing depressive symptoms. This study investigates the underexplored cognitive mechanisms of clozapine-induced OCS, particularly focusing on dysfunctional checking behavior.

**Methods:**

Clinical and cognitive profiles of OCS and their relationship to dysfunctional checking were investigated using a novel checking paradigm (image verification task or IVT) in four groups: clozapine-treated schizophrenia patients with clozapine-induced OCS (SCZ-OCS, n = 21) and without (SCZ-only, n = 15), patients with obsessive-compulsive disorder (OCD, n = 32) and IQ-matched healthy volunteers (HV, n = 30).

**Results:**

Only SCZ-OCS patients showed a distinctive pattern of dysfunctional checking on the IVT. Compared with SCZ-OCS, SCZ-only patients exhibited functional checking while having equivalent deficits in executive cognition, clozapine dose, and treatment duration, though with less severe positive and depressive symptoms. In SCZ-OCS, dysfunctional checking was positively correlated with clozapine dose and working memory performance. By contrast, OCD patients’ checking was positively related to intolerance of uncertainty. Checking in the OCD and SCZ-OCS groups was positively correlated with YBOCS-compulsion.

**Conclusion:**

This study is the first to compare the distinct cognitive and clinical profiles of SCZ-OCS, SCZ-only, and OCD, with a focus on checking behavior, a major symptom in clozapine-treated patients. We introduced a novel and sensitive measure for checking, which showed dysfunctional checking only in SCZ-OCS patients treated with clozapine. These findings indicate that a subset of patients with schizophrenia with more severe positive symptoms and cognitive deficits are especially susceptible to OCD symptoms when treated with clozapine.

## Introduction

Obsessive compulsive symptoms (OCS), such as intrusive thoughts and the compulsion to perform ritualistic behaviors, develop in a significant proportion of chronic schizophrenia patients treated with clozapine (Fernandez-Egea et al., 2018, Schirmbeck & Zink, 2012, Grover et al., 2015). These symptoms are associated with a range of negative outcomes, including reduced social functioning (Tonna et al., 2015), increased positive and depressive symptoms (Biria et al., 2019; Parkin et al., 2023), and a greater risk of suicide (Szmulewicz et al., 2015). However, despite the high prevalence of this comorbidity and its negative consequences, no studies have yet characterized the cognitive mechanisms underpinning their development after starting the treatment with clozapine.

Clozapine is an atypical or second-generation antipsychotic, typically utilized to manage refractory schizophrenia. It binds to a large number of receptors (Stahl, 2000), acting as an antagonist especially at 5-HT2A, 5-HT1A, and 5-HT2C- receptors, as well as dopamine (especially D2) receptors (Meltzer & Huang, 2008). Both the incidence and severity of OCS among patients with chronic schizophrenia has been positively associated with clozapine dose and/or plasma levels (Biria et al., 2019; Fernandez-Egea et al., 2024; Gürcan et al., 2021; Kim et al., 2020; Lin et al., 2006; Mukhopadhaya et al., 2009; Reznik et al., 2004; Schirmbeck et al., 2011). Moreover, duration of clozapine treatment is also positively associated with symptom severity – while 25% of patients exhibit excessive checking behavior after 5 years of treatment, this rises to over 50% after 10 years (Fernandez-Egea et al., 2018). Excessive checking is the most commonly reported obsessive compulsive symptom in both pure OCD patients (Ruscio et al., 2010) and schizophrenia patients with OCS (Fernandez-Egea et al., 2018, Grover et al., 2015). However, the factors causing excessive checking behavior remain unclear.

Previously, checking in schizophrenia has mainly been studied with self-reported measures such as obsessive-compulsive inventory (OCI; Foa et al., 1998) and, to our knowledge, has never been compared in groups of patients with and without OCS. However, an objective means of assessing checking under different environmental demands in these groups as well as OCD is required. A variety of behavioral tasks has been used to investigate checking in OCD patients (Clair et al., 2013; Jaafari et al., 2013; Rotge et al., 2015), but these tasks have not considered the *functionality* of checking. Checking is functional if it improves the monitoring of performance but is dysfunctional and superfluous if it does not. For this purpose, we used in this study our novel IVT task (Biria et al., 2024), which has been designed to capture this distinction under a variety of conditions leading to anxiety including high uncertainty and reinforcement contingencies such as punishment.

A comparison, therefore, between checking performance across the two schizophrenia groups (SCZ-OCS and schizophrenia without OCS) with equivalent dose of clozapine and treatment duration and OCD patients is required. Previously, OCD and SCZ-OCS patients have been differentiated in terms of their cognitive flexibility deficits (Chamberlain et al., 2021; Patel et al., 2010; Schirmbeck et al., 2013). While working memory deficits are prominent in schizophrenia patients (Bowie & Harvey, 2006; Forbes et al., 2009; Goldberg & Green, 2002; Lee & Park, 2005) and could possibly play a role in checking behavior, they are less commonly reported in OCD patients as a cause for checking (Greisberg & McKay, 2003; Kalenzaga et al., 2020; Olley et al., 2007; Persson et al., 2021; Woods et al., 2002).

We aimed to test the following hypotheses among four groups: patients with OCD, clozapine-treated schizophrenia patients with (SCZ-OCS; with OCS starting only after the clozapine treatment) and without OCS (SCZ-only), and healthy volunteers (HV):

**H1**: OCD and SCZ-OCS patients will show dysfunctional checking (i.e., checking that is dysfunctional, which does not improve performance, hence superfluous), particularly under conditions of high uncertainty and punished checking.

**H2**: Aspects of executive cognition, such as cognitive flexibility and working memory, will be more impaired among subjects with SCZ-OCS compared to other groups and may be related to their excessive checking behavior.

**H3**: Clozapine dosage, treatment duration, and schizophrenia symptoms (positive and depressive symptoms) will be related to checking in SCZ-OCS but not the SCZ-only group.

Overall, testing these hypotheses will enhance our understanding of the significance of checking symptoms in schizophrenia as another aspect of executive cognition contributing to functionality in everyday life.

## Methods

### Participants

Participants were 30 healthy volunteers, 32 OCD patients, and 38 clozapine-treated schizophrenia patients. Of the latter, 23 exhibited OCS, and were enrolled in the SCZ-OCS group, while 15 participants did not exhibit OCS (the SCZ-only group). All participants were fluent in English, possessed normal or corrected-to-normal vision and were matched for age and verbal IQ. The OCD and healthy groups were also matched for gender. Table 1 shows the demographic and clinical characterization of all groups. This study was approved by the East of England - Cambridge South Research Ethics Committee (REC 16/EE/0465) for OCD and healthy volunteers and the Cambridge and Peterborough NHS Foundation Trust (REC 18/EE/0073) for patients with schizophrenia. All volunteers gave written informed consent before beginning the testing and received monetary compensation for taking part in the study.

**Table 1.** This table depicts the mean ± standard deviations of all demographics, clinical and cognitive measures, and post-hoc comparisons for all the groups

	HV (M ± SD)	OCD (M ± SD)	SCZ-OCS (M ± SD)	SCZ-only (M ± SD)	F/t	p	D	95% CI	ranking
**Demographic information**									
N	30	32	21	15					
Age (Years)	40.37 ± 12.21	35.78 ± 12.99	44.10 ± 10.54	46.73 ± 11.22	14.49	< .001	0.10^ηp2^	[0.04, 0.15]	[SCZ-only > HV = SCZ-OCS > OCD]
Gender (M:F)	14:16	13:19	17:04	13:02	60.65^X2^	< .001	0.39^Phi^	[0.19, 0.58]	NA
Verbal IQ (NART)	116.44 ± 6.03	116.96 ± 4.35	117 ± 5.96	113.77 ± 9.21	3.86	0.01	0.31^ηp2^	[0.002, 0.066]	[HV = OCD = SCZ-OCS > SCZ-only]
Education (Years)	17.17 ± 3.15	15.63 ± 3.10	NA	NA	−1.91	0.06	0.12^ηp2^	[0.055, 0.18]	[HV > OCD]
**Clinical measures**									
YBOCS Obsession	NA	10.75 ± 2.71	7.57 ± 3.25	NA	3.71	< .001	1.08	[0.48, 1.68]	[OCD > SCZ-OCS]
YBOCS Compulsion	NA	10.56 ± 3.38	8.81 ± 4.09	NA	8.46^U^	0.02	NA	[0.41, 4]	[OCD > SCZ-OCS]
YBOCS total score	NA	21.31 ± 5.44	15.33 ± 4.56	NA	8.32	< .001	1.16	[0.87, 1.46]	[OCD > SCZ-OCS]
Depression (MADRS)	2.33 ± 3.91	9.44 ± 7.89	9.95 ± 4.05	5.33 ± 6.00	41.71^w^	< .001	0.24^ηp2^	[0.17, 0.30]	[OCD = SCZ-OCS > SCZ-only > HV]
State anxiety (STAI-S)	26.93 ± 6.14	40.06 ± 11.10	43.38 ± 11.58	38.40 ± 11.56	55.41^w^	< .001	0.30^ηp2^	[0.22, 0.36]	[OCD = SCZ-OCS > SCZ-only > HV]
Trait anxiety (STAI-T)	32.33 ± 8.8	54.61 ± 10.25	51.33 ± 8.93	42.60 ± 12.43	117.01^w^	< .001	0.47^ηp2^	[0.40, 0.53]	[OCD = SCZ-OCS > SCZ-only > HV]
IOU	47.17 ± 20.11	74.50 ± 22.53	76.09 ± 23.28	59.93 ± 16.94	45.47^w^	< .001	0.26^ηp5^	[0.18, 0.32]	[OCD = SCZ-OCS > SCZ-only > HV]
OCI (total score)	8.37 ± 9.84	58.62 ± 28.18	53.14 ± 32.57	24.60 ± 16.63	112.96^w^	< .001	0.46^ηp2^	[0.39, 0.52]	[OCD = SCZ-OCS > SCZ-only > HV]
OCI-washing	1.47 ± 2.71	11.09 ± 10.3	7.10 ± 7.12	2.93 ± 3.19	43.52^w^	< .001	0.25^ηp2^	[0.17, 0.31]	[OCD > SCZ-OCS > SCZ-only > HV]
OCI-checking	1.47 ± 2.11	11.81 ± 7.69	12.48 ± 8.08	5.27 ± 3.81	82.04^w^	< .001	0.38^ηp2^	[0.31, 0.45]	[OCD = SCZ-OCS > SCZ-only > HV]
OCI-doubting	0.40 ± 0.92	5.56 ± 3.14	4.81 ± 3.32	1.73 ± 1.30	109.08^w^	< .001	0.45^ηp2^	[0.38, 0.51]	[OCD = SCZ-OCS > SCZ-only > HV]
OCI-ordering	1.80 ± 2.73	6.41 ± 4.34	5.38 ± 4.69	2.60 ± 3.24	35.71^w^	< .001	0.21^ηp2^	[0.14, 0.28]	[OCD = SCZ-OCS > SCZ-only = HV]
OCI-neutralizing	0.83 ± 1.19	7.25 ± 4.49	7.24 ± 4.66	3.07 ± 3.24	83.31^w^	< .001	0.39^ηp2^	[0.31, 0.45]	[OCD = SCZ-OCS > SCZ-only > HV]
OCI- hoarding	1.07 ± 1.55	2.97 ± 2.98	3.90 ± 2.74	3.67 ± 3.40	169.11^w^	< .001	0.15^ηp2^	[0.09, 0.21]	[OCD = SCZ-OCS = SCZ-only > HV]
OCI-obsessing	1.27 ± 1.81	13.53 ± 6.91	11.86 ± 7.87	5.27 ± 5.13	108.44^w^	< .001	0.45^ηp2^	[0.38, 0.51]	[OCD > SCZ-OCS > SCZ-only > HV]
Smoker (Yes:No)	0:30	0:32	6:15	5:10	21.54X^2^	< .001	0.47^Phi^	[0.37, 0.57]	NA
**Clinical measures: schizophrenia groups only**									
Clozapine dose	NA	NA	309.52 ± 91	323.33 ± 111	0.39	0.69	0.13	[−0.53, 0.79]	[SCZ-OCS = SCZ-only]
Clozapine duration	NA	NA	17.63 ± 7.72	18.92 ± 7.08	0.45	0.65	0.16	[−0.55, 0.89]	[SCZ-OCS = SCZ-only]
PANSS-Positive (P1–7)	NA	NA	14.52 ± 4.77	10.67 ± 4.59	236^U^	0.01	NA	[1, 7]	[SCZ-OCS = SCZ-only]
PANSS-Negative(N1–7)	NA	NA	15.00 ± 7.16	15.33 ± 7.83	165^U^	0.82	NA	[−6, 4]	[SCZ-OCS > SCZ-only]
PANSS-General(G1–16)	NA	NA	29.48 ± 6.63	24.47 ± 6.49	217^U^	0.05	NA	[−0.00002, 9]	[SCZ-OCS > SCZ-only]
AIMS	NA	NA	0.81 ± 0.39	1 ± 0.39	175^U^	0.51	NA	[−3.30, 0.99]	[SCZ-OCS = SCZ-only]

Acronyms: HV = healthy volunteers, OCD = obsessive compulsive disorder, SCZ-OCS = schizophrenia with OCS, SCZ-only = schizophrenia without OCS*, NART* National Adult Reading Test, *YBOCS* Yale-Brown Obsessive Compulsive Scale, *MADRS* Montgomery-Asberg Depression Rating Scale, *STAI-S* State Trait Anxiety Inventory-State, *STAI-T* State Trait Anxiety Inventory-Trait, *IOU* Intolerance Of Uncertainty, *OCI* Obsessive Compulsive Inventory, *PANSS* Positive and Negative Symptoms Scale, *AIMS* Abnormal Involuntary Movement Scale, F/t F-test and t-test were calculated for variables that were available for four versus two groups respectively, X^2^ Chi-Square test for categorical data, U and W stand for Mann-Whitney U and Welch’s ANOVA tests respectively, in case of non-normal/inhomogeneous data, ηp^2^ partial eta-square (a measure of effect size for the U test), Phi a measure of effect size for the X^2^ test, d Cohen’s d, CI Confidence Interval for the effect sizes. All tests were two-sided except for OCI, STAI-S, STAI-T, and IOU where one-sided tests were used. For the U tests, where no effect size could be calculated, the 95% U test CI is reported instead.

Presence of an OCD or schizophrenia diagnosis was confirmed through clinician assessment with reference to DSM-IV criteria. All OCD patients had a primary diagnosis of OCD and no other comorbid Axis-I mental disorders. OCD and SCZ-OCS patients were recruited if they scored >7 on the Yale- Brown Obsessive Compulsive Scale (YBOCS, Goodman et al., 1989) and > 42 on the obsessive-compulsive inventory (Foa et al., 1998). SCZ-only patients were included if after 5 years of treatment on clozapine they scored <3 on the YBOCS, and a total score of <42 on the OCI. Additionally, any schizophrenia patients with a history of OCD (either successfully treated or with ongoing OCS) were not recruited for this study. Healthy controls had no current or past psychiatric disorders as determined by a screening interview including the Mini International Neuropsychiatric Interview (MINI; Sheehan et al., 1998), the Montgomery-Asberg Depression Rating Scale (MADRS; Montgomery & Asberg, 1979), and the OCI (< 42). For all participants, excessive drug or alcohol use, neurological deficits, or head injury were exclusion criteria. Both groups of schizophrenia patients (SCZ-OCS and SCZ-only) received comparable clozapine doses and had undergone comparable duration of treatment (minimum of 5 years). These criteria were necessary to avoid confounding effects of treatment on clinical or cognitive symptoms between the two groups. Additionally, a treatment duration of longer than 5 years was selected based on the study by Fernandez-Egea et al. (2018) to allow enough time for the development of OCS. As apparent from the above criteria, the inclusion of the schizophrenia patients in our study was particularly strict. At the time of this study, the clozapine clinic research database contained 238 clozapine-treated patients. However, the majority of these patients had some exclusion criteria in terms of treatment duration or other potential confounders resulting in the small sample sizes of 21 and 15 for our two schizophrenia groups.

Among the SCZ-OCS patients, 3 had comorbidities with depression, 1 had a diagnosis of Asperger syndrome, 1 schizoaffective disorder and 2 dyslexia. In the SCZ-only group, 1 had an emotionally unstable personality disorder, and 2 had comorbid generalized anxiety disorder. Thirteen SCZ-OCS and three SCZ-only patients were treated solely with clozapine, the rest were on a combination of medications. In the OCD group, all but 9 patients were medicated, majority of whom treated with SSRIs. See Supplementary Materials for more details on medication. None of the healthy controls were medicated. Besides 5 schizophrenia patients without OCS, and 7 with OCS, no one else including the healthy and OCD groups was a smoker. Finally, two SCZ-OCS patients were excluded from the study due to unreliable performance and an epilepsy diagnosis after the data collection leaving a sample of 21 SCZ-OCS patients.

## Clinical assessments

All participants were assessed using (1) obsessive-compulsive inventory (Foa et al., 1998), a 42-item self-report scale assessing OCS using 6 subscales (washing, checking, ordering, hoarding, obsessional thinking, and mental neutralizing) to produce a score between 0 and 126. Higher scores indicate greater symptomology; (2) State/Trait Anxiety Questionnaire (Spielberger et al., 1983), a 20-item self-report scale assessing anxiety; (3) National Adult Reading Test (Nelson & Willison, 1982), an estimate of verbal intelligence; (4) Intolerance of Uncertainty Scale (Buhr & Dugas, 2002), a 27-item self-report scale assessing participants’ response to uncertainty in daily life; and (5) a forward and backwards digit task from the WAIS-III, assessing verbal working memory (Wechsler, 1997). The following additional assessments were undertaken for participants with schizophrenia: (1) The Positive and Negative Syndrome Scale (PANSS; Kay et al., 1987), which is a clinician-administered 30-item tool exploring positive (7 items), negative (7 items) and general symptoms of schizophrenia (16 items); (2) The Abnormal Involuntary Movement Scale (AIMS; Guy, 1976), which measures involuntary movements. Finally, clozapine dosage, treatment duration, and smoking habits were also collected for these participants.

## Behavioral measures

### Cognitive flexibility

The CANTAB Intra-Extra Dimensional Set Shift (Owen et al., 1991; Roberts et al., 1988) was used to assess cognitive flexibility, more specifically set-shifting ability. This 7-minute task tests for rule acquisition, reversal learning, and attentional set shifting. It features visual discrimination between color-filled shapes and white lines, involving shifting and flexibility of attention. The test is well validated in individuals experiencing OCD (Chamberlain et al., 2007; Vaghi et al., 2017) and schizophrenia (Leeson et al., 2009; Pantelis et al., 1999) and is a computerized analogue of the Wisconsin Card Sorting test. It was administered on a touch-screen tablet.

### Spatial and verbal working memory

Participants were additionally tested on CANTAB self-ordered Spatial Working Memory (SWM) task (Owen et al., 1990). In this 4-minute task, participants performed a sequence of responses on a touch sensitive screen tablet to detect the location of ‘reward’ tokens. It tests spatial working memory and executive functioning. In this paper, we only report the SWM between errors (SWMBE), which are the number of times someone revisits a box they had already visited. An additional test was used to assess the capacity of verbal working memory: the forward and backwards digit task from the WAIS-III (Wechsler, 1997). Participants had to repeat series of digits of increasing length, read by the researcher. Digit span forward consists of 2 levels within of 8 sets of digits, whereas digit span backwards has 2 sets of 7 digits. If a participants made a mistake on both levels within a set, the test would stop. With these 2 tasks, we covered measurements of working memory and executive functioning.

### Checking behavior

**Figure 1.**
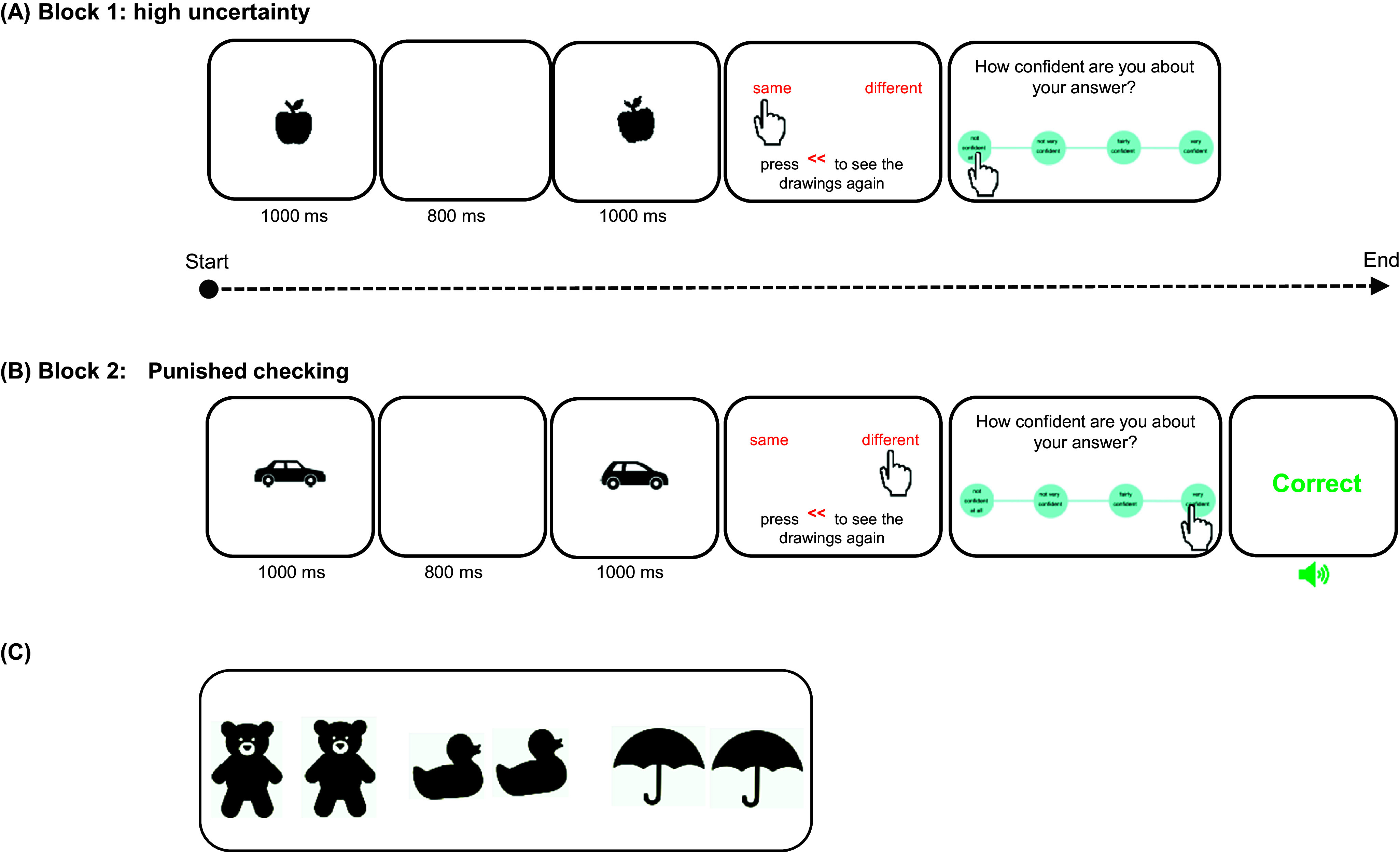
A schematic representation of the Image verification task reproduced from Biria et al., 2024. Participants observed two black and white drawings sequentially. The task was to compare them and decide if they are the same or different. There was an opportunity to check the images, before giving a response, by pressing on the red **<<** sign. After each answer, there was a 4-choice confidence rating scale: ‘not confident at all’, ‘not very confident’, ‘fairly confident’, and ‘very confident’. Each stimulus presentation lasted 1 second with an 800 ms interstimulus interval. The remaining frames remained on the screen until an answer was given. (A) Block 1 is the high uncertainty block, which provided no feedback. In this example, the stimuli differ in angle. (B) Block 2 punished checking by adding one trial for each check (that is every time the participant pressed the checking sign on the screen) and feedback was provided for all trials to reduce uncertainty. Wrong and correct answers were followed by both visual (red and green, respectively) and auditory feedback (aversive and uplifting sound, respectively). Here, two different objects are depicted. (C) Three examples of stimuli used in this task. From left to right: 2 bears (the right bear is bigger), 2 ducks (the right duck is more crooked), and 2 umbrellas (the umbrellas are exactly the same).

Checking behavior was assessed using a novel experimental paradigm – the image verification task (IVT), administered via a touchscreen XPS 15.6” DELL laptop. IVT is a conceptually simple task, yet perceptually difficult enough to increase doubt and checking. At each trial, participants observed two black and white drawings of objects presented in rapid succession and were asked to determine if the two items were identical or different in size, shape or angle (see Figure 1). Optimizing both accuracy of answers and overall speed were instructed as the main task goals. The IVT utilized in this study differed slightly from the task recently published in Biria et al., 2024, where the sole instruction was to improve accuracy of answers without the need to consider the overall speed. In this study, we chose to incorporate an emphasis on speed as a measure of goal-directed behavior, encouraging the use of information from checking, to enable good performance, avoiding excessive checking that could detrimentally impact overall task speed and performance. Before making their decision and providing their answer, participants had the opportunity to review the images as many times as they liked: an explicit measure of checking. Each object was presented for 1 second, separated by an 800 ms white image interstimulus interval. After making a choice, participants rated their confidence about their answer on 4-choice scale ranging from ‘not confident at all’ to ‘very confident’ (see Figure 1A). The task comprised two blocks of 45 trials each, presented sequentially, assessing different aspects of cognition and perceptual decision-making that may be implicated in checking (specifically uncertainty and punishment). The first block involved high uncertainty and no feedback on performance (Figure 1A) while the second block punished checking with an extra trial every time participants checked and provided performance feedback by seeing the words ‘Correct’ in green or ‘Wrong’ in red on the screen (Figure 1B).

## Data analysis

### Clinical assessment, cognitive flexibility, and spatial working memory

A series of analyses of variances (ANOVAs) was performed to compare clinical and cognitive measures, as well as performance on IED and SWM tasks between groups. The Welch’s ANOVA test was used when a non-parametric test was required. Multiple comparison tests were performed for variables that displayed a significant variation between groups. For variables available only in two groups, an independent sample t-test was used in case of a normal distribution, otherwise a Mann–Whitney U test was applied.

### Checking behavior

Correlation between checking rates and accuracy of performance was used to inspect the functionality of checking behavior. Associations between IVT performance and clinical measures and questionnaire scores were tested using Pearson’s r correlation coefficient, or Spearman’s r if data were non-parametric. The Williams’s Test (z) was used to compare two independent correlations between groups. To compare the clozapine dose between the two schizophrenia groups, an ANCOVA (analysis of covariance) was used with number of cigarettes smoked and gender as covariate. Finally, checking was compared between groups using three orthogonal contrasts performed using repeated-measures ANOVAs. The contrasts were between (1) healthy volunteers (HV) vs patients (OCD and schizophrenia groups), (2) OCD vs schizophrenia (SCZ-OCS + SCZ-only), and finally, (3) SCZ-OCS vs SCZ-only groups. Dependent variables in these analyses included average checking rates, accuracy of answers in percentage, and participant confidence ratings. If significant main effects were identified, post hoc multiple comparisons were performed using a Bonferroni correction. SPSS version 29 was used to perform the repeated-measures ANOVAs, while the rest of the analyses were performed using Python version 3.7.6 and RStudio version 4.2.3.

### Code availability

The R code and output used for the contrast analyses are provided in the Supplementary Materials.

## Results

### Demographics and clinical assessments

Table 1 displays the ANOVA results comparing all demographic, clinical assessments between groups, with their corresponding mean and SD. The multiple comparisons are presented in Table S1 in the Supplementary Materials.

### Cognitive flexibility and working memory

Table 2 depicts the group comparisons, averages, and standard deviations for the cognitive and behavioral measures on the CANTAB IED and SWM tasks, digit span task, and the IVT. The schizophrenia groups exhibited poorer performance on all three cognitive measures as compared to OCD and HV participants. On the SWM, schizophrenia patients revisited boxes they had already visited (SWMBE) significantly more than the remaining groups (*p* < 0.05). Similar to the SWM findings, schizophrenia groups performed worse on digit span backward, remembering fewer digits (*p* < 0.05). However, the groups did not differ on digit span forward (*p* > 0.05). On the IED task, schizophrenia patients also demonstrated worse performance at all task stages (*p* < 0.01). More specifically, they completed less stages on the task and made more errors on the extra-dimensional set shift stage (EDS) of the CANTAB IED task, a critical measure of cognitive flexibility. Whereas the number of errors measured at the earlier stage of PRE-EDS was worse in SCZ-OCS compared to the SCZ-only group, both schizophrenia groups were worse than HV and OCD groups. Table 2 defines significant performance differences among the groups. Table.S1 in Supplementary Materials shows the independent multiple comparison tests for all significant group differences in Table 2.

**Table 2.** Shows the mean ± standard deviations for working memory, cognitive flexibility, and behavioral measures on the IVT for all groups

	HV (M ± SD)	OCD (M ± SD)	SCZ-OCS (M ± SD)	SCZ-only (M ± SD)	F/W	p	d	95% CI	ranking
**Working memory**									
Digit span forward	10.4 ± 1.93	11.68 ± 3.11	10.24 ± 2.44	10.73 ± 2.53	7.18^w^	0.16	0.05^ηp2^	[0.01, 0.09]	[OCD > HV = SCZ-OCS = SCZ-only]
Digit span backwards	7.17 ± 2.09	8.13 ± 2.62	6.05 ± 1.90	6.07 ± 2.28	18.69^w^	< .05	0.12^ηp2^	[0.06, 0.18]	[OCD > HV > SCZ-OCS = SCZ-only]
SWMBE	7.46 ± 8.81	9.58 ± 9.37	15.38 ± 8.93	14.67 ± 9.11	16.48	< .001	0.11^ηp2^	[0.05, 0.17]	[OCD = HV < SCZ-OCS = SCZ-only]
**Cognitive flexibility**									
IED completed stages	8.66 ± 1.32	8.58 ± 0.79	7.10 ± 2.72	8.27 ± 0.93	19.31^w^	0.01	0.13^ηp2^	[0.07, 0.19]	[HV = OCD = SCZ-only > SCZ-OCS]
IED PRE-EDS errors	7.66 ± 6.26	6.03 ± 1.9	14.62 ± 9.76	9.33 ± 4.01	36.29^w^	< .001	0.22^ηp2^	[0.15, 0.28]	[OCD = HV < SCZ-only < SCZ-OCS]
IED EDS errors	6.24 ± 6.98	9.10 ± 9.45	16.14 ± 10.79	15.33 ± 10.05	21.15^w^	< .001	0.16^ηp2^	[0.09, 0.22]	[OCD = HV < SCZ-only = SCZ-OCS]
**IVT measures**									
Confidence_B1	3.36 ± 0.31	3.23 ± 0.35	3.22 ± 0.47	3.08 ± 0.36	2.09	0.1	0.06^ηp2^	[0.00, 0.15]	[HV = OCD = SCZ-OCS = SCZ-only]
Accuracy_B1	78.70 ± 4.81	78.47 ± 7.36	69.86 ± 7.74	71.13 ± 8.64	10.52	< .001	0.05^ηp2^	[0.09, 0.37]	[HV = OCD > SCZ-OCS = SCZ-only]
Checking_B1	19.90 ± 13.80	21.03 ± 15.66	12.00 ± 14.58	11.33 ± 10.99	2.91	0.03	0.08^ηp3^	[0.00, 0.18]	[HV = OCD = SCZ-OCS = SCZ-only]
Confidence_B2	3.10 ± 0.31	2.93 ± 0.50	2.96 ± 0.60	2.94 ± 0.50	0.82	0.48	0.02^ηp4^	[0.09, 0.08]	[HV = OCD = SCZ-OCS = SCZ-only]
Accuracy_B2	77.13 ± 7.72	76.09 ± 8.82	69.05 ± 9.32	69.60 ± 7.14	5.89	< .001	0.05^ηp5^	[0.00, 0.004]	[HV = OCD ≥ SCZ-only = SCZ-OCS]^*^
Checking_B2	5.03 ± 8.26	5.31 ± 7.19	6.10 ± 7.89	5.80 ± 10.39	0.08	0.97	0.15^ηp6^	[0.03, 0.27]	[HV = OCD = SCZ-OCS = SCZ-only]

Acronyms: HV = healthy volunteers, OCD = obsessive compulsive disorder, SCZ-OCS = schizophrenia with OCS, SCZ-only = schizophrenia without OCS*, SWMBE* Spatial Working Memory Between Errors (the number of times the subject incorrectly revisits a box in which a token has previously been found), *IED* Intra-Extra Dimensional Set Shift, ED extradimensional shift, IVT = Image Verification Task, B1 = block 1, B2 = block 2, F/W F-test or Welch’s ANOVA tests respectively, in case of non-normal/inhomogeneous data, ηp^2^ partial eta-square as a measure of effect size for the ANOVA tests, 95% CI Confidence Interval for the effect sizes. All tests were two-sided. *: for Accuracy_B2, the following was the exact ranking: HV = OCD, OCD = SCZ-only, OCD > SCZ-OCS, HV > SCZ-only and SCZ-OCS.

### Functional checking behavior

**Figure 2.**
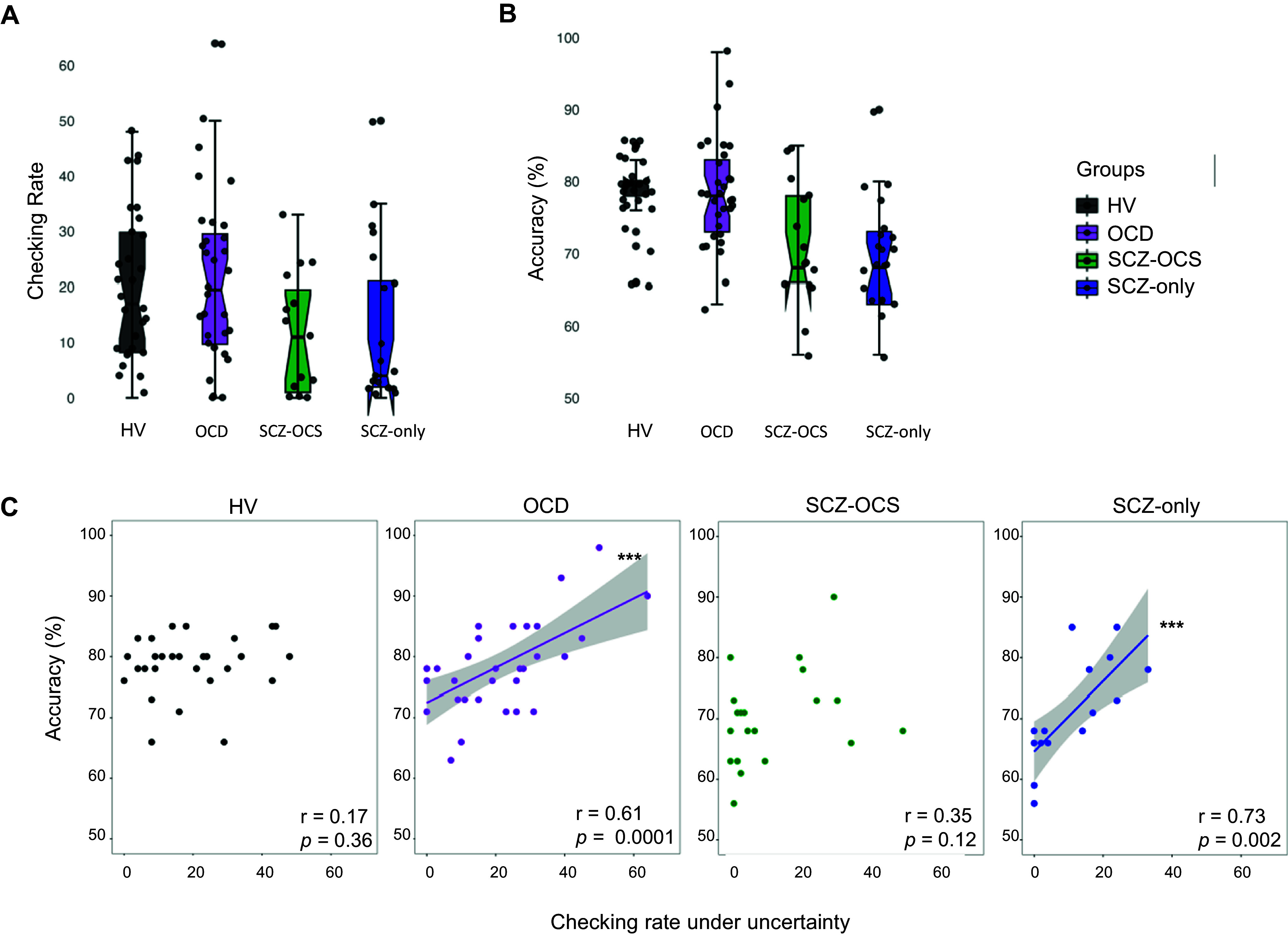
IVT performance under high uncertainty (A) showing the checking rate, and (B) the accuracy of answers. (C) depicts the relationship between checking and percentage accuracy under high uncertainty (block 1) as a measure of functionality of checking in healthy volunteers (HV; black), OCD (purple), SCZ-OCS (green), and SCZ-only (blue) patients. The line of best fit is shown with the 95% confidence intervals for the regression estimate in translucent bands around the regression lines. The r indicates Pearson correlation coefficient for which two-tailed tests were used. Acronyms: HV = healthy volunteers, OCD = obsessive compulsive disorder, SCZ-OCS = schizophrenia with OCS, SCZ-only = schizophrenia without OCS. *** *p* < 0.001.

Table 2 shows the descriptive statistics for all IVT task measures across groups. Response accuracy and checking were significantly correlated in OCD (r = 0.61, *p* < 0.001) and SCZ-only (r = 0.73, *p* < 0.001) groups in block 1 under high uncertainty. This block had the highest checking rate for these groups. This was not the case for the SCZ-OCS group, who checked less and less effectively. In healthy subjects, however, the high levels of accuracy depended on less checking (in comparison e.g. with OCD patients), which may explain the lack of correlation between checking and performance. The difference between these correlations in the two schizophrenia groups was significant (z = 2.079, *p* < 0.01), and OCD and healthy volunteers (z = 1.93, p = 0.02), but not between OCD and SCZ-OCS (*p* > 0.05). Figure 2 depicts this relationship in all groups. Table S2 shows the descriptive statistics for all task performance measures in all groups and over all blocks.

**Figure 3.**
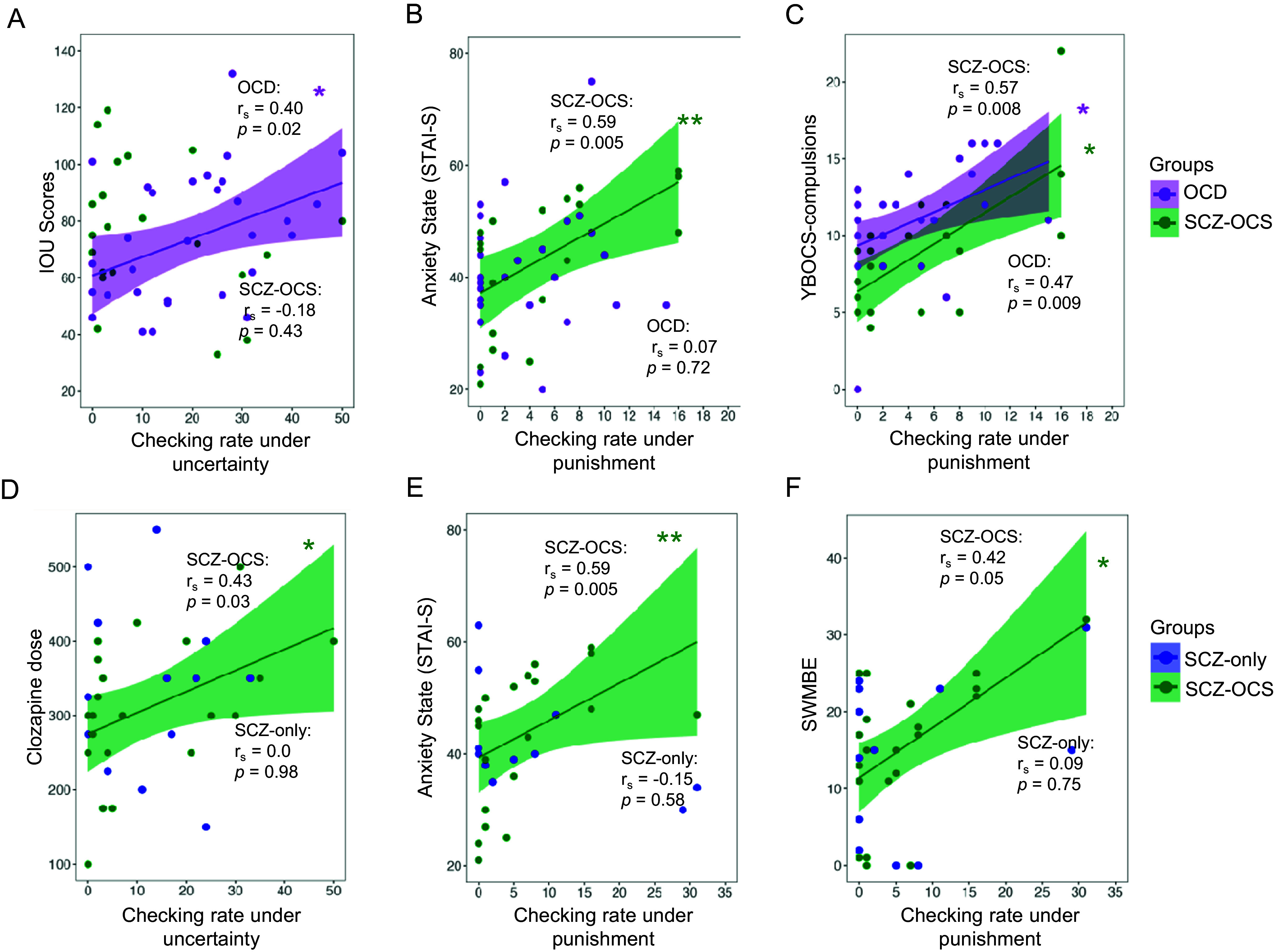
Relationship between checking and clinical measures. The upper panel shows the comparison between OCD and SCZ-OCS groups, whereas the lower panel compares the two schizophrenia groups. The following relationships are depicted: 1) between OCD (purple) and SCZ-OCS (green) patients: (A) checking under uncertainty and intolerance of uncertainty scores (IOU), (B) checking under punishment and anxiety state (STAI-S), (C) checking under punishment and YBOCS compulsion; 2) between the SCZ-OCS (green) and SCZ-only (blue) patients: (D) checking under uncertainty and clozapine dose, (E) checking under punishment and anxiety state (STAI-S), (F) checking under punishment and CANTAB SWMBE. The line of best fit is shown with the 95% confidence intervals for the regression estimate in translucent bands around the regression lines. The r_s_ indicates Spearman correlation coefficient for which two-tailed tests were used. The asterisks indicate significance and their color correspond to the group they are representing. Acronyms: HV = healthy volunteers, OCD = obsessive compulsive disorder, SCZ-OCS = schizophrenia with OCS, SCZ-only = schizophrenia without OCS, *IOU* Intolerance Of Uncertainty, *STAI-S* State Trait Anxiety Inventory-State, *YBOCS* Yale-Brown Obsessive Compulsive Scale, *SWMBE* Spatial Working Memory Between Errors (the number of times the subject incorrectly revisits a box in which a token has previously been found). * *p* < = 0.05, ** *p* < 0.01.

### Relationship between checking under uncertainty and clinical measures

Only in OCD patients, was functional checking under uncertainty (block 1) positively correlated with intolerance of uncertainty scores (r_s_ = 0.40, *p* = 0.02), but this was not the case in other groups. Clozapine dose was significantly correlated with checking under uncertainty (block 1) only in the SCZ-OCS group (SCZ-OCS: r_s_ = 0.44, *p* = 0.04; SCZ-only: r_s_ = 0, *p* = 0.98). When accounting for cigarette use and gender, known modulators of clozapine metabolism and plasma levels (Mayerova et al., 2018), this partial correlation remained very similar (SCZ-OCS: r_s_ = 0.428, *p* < 0.05; SCZ-only: r_s_ = 0.14, *p* = 0.31).

### Relationship between punished checking, clinical measures, and SWM

For punished checking, two OCD patients who were outliers for checking (>2SD from the mean) in the second block were excluded from this part of the analysis. Both these patients were checkers according to their OCI-checking sub-scores and after excluding them, the YBOCS compulsion score in OCD patients was correlated with the punished checking rate in block 2 (r_s_ = 0.46, *p* < 0.01). A similar relationship between punished checking and YBOCS compulsion sub-scores was found in the SCZ-OCS group (r_s_ = 0.51, *p* < 0.01), who additionally showed a positive correlation between punished checking and state anxiety (r_s_ = 0.60, *p* = 0.004). There were no such correlations between punished checking and state anxiety in SCZ-only group (r = −0.15, *p* = 0.29).

Previous findings of association between treatment duration and checking behavior (Fernandez-Egea et al., 2018) were only replicated in SCZ-OCS patients who showed a non-significant trend (SCZ-OCS: r_s_ = 0.40, *p* = 0.09; SCZ-only: r_s_ = 0.20, *p* = 0.54). The latter non-significant finding could be due to the fact that only patients who were treated longer than 5 years on clozapine were recruited for this study as opposed to the patient cohorts in Fernandez-Egea et al. (2018). There were no correlations between PANSS sub-scores and IVT checking in either schizophrenia groups in block 1. However, in block 2, under punished checking, there were positive trends only in SCZ-OCS group between all PANSS sub-scores and checking (Positive: r = 0.39, *p* = 0.08; Negative: r = 0.36, *p* = 0.10; General: r = 0.49, *p* = 0.02). Moreover, SWM deficit (SWMBE: revisiting a previously visited box again) was also marginally correlated with checking under punishment (r_s_ = 0.42, *p* = 0.05) and significantly correlated with clozapine dose (r_s_ = 0.37, p = 0.01) in the SCZ-OCS group but not among SCZ-only patients (r_s_ = 0.09, *p* = 0.38; r_s_ = −0.016, *p* = 0.47, respectively for SWMBE and clozapine dose), indicating the role of working memory deficit not only for checking but also in association to clozapine dose in SCZ-OCS patients.

### Contrast analysis and IVT group differences

Finally, we analyzed three contrasts (C1, C2, and C3) comparing the checking, confidence rating, and accuracy of answers between C1) HV vs patients, C2) OCD vs schizophrenia, and C3) SCZ-OCS vs SCZ-only groups for block 1 (high uncertainty, no feedback) and block 2 (punished checking, feedback). Supplementary Figure S1 depicts performance on the IVT for all three contrasts. Across all contrasts checking and confidence reduced from block 1 (high uncertainty) to block 2 (punished checking) in all groups. There were group differences in C1 (Supplementary Figure S1.A) with patients checking less and being less confident than healthy volunteers, and in C2 (Supplementary Figure S1.B) with OCD patients checking more and being more accurate than both schizophrenia groups. There were no group differences in C3 (Supplementary Figure S1.C) between the two schizophrenia patients. The R code and the output of all three contrasts are shown in the Supplementary Materials. Tables S2 and S3 in the Supplementary Materials, respectively, show the descriptive statistics for all measures on the IVT and the independent multiple comparison tests for all groups.

## Discussion

OCS are prevalent in a significant proportion of schizophrenia patients treated with clozapine. Fernandez-Egea et al. (2024) reported this rate to be approximately 38%, although in our less representative sample, 58% of schizophrenia patients experienced OCS 5 years after beginning clozapine treatment.

In this study, we compared the cognitive, clinical, and behavioral characteristics of OCS in schizophrenia patients experiencing clozapine induced OCS, in comparison with schizophrenia patients without OCS, OCD patients and healthy control subjects.

The IVT, a novel behavioral paradigm, was used to study dysfunctional checking for the first time in patients with schizophrenia in a laboratory setting. As expected, healthy controls exhibited high levels of accuracy. While checking in this group was not associated with improved performance, this finding is likely explained by a ceiling effect as they consistently exhibited optimal perceptual accuracy. The SCZ-OCS group, however, exhibited dysfunctional checking under uncertainty as their checking behavior was not related to their accuracy of perceptual decision-making. OCD and SCZ-only patients, however, showed functional checking by using the information from checking to monitor the accuracy of their performance. All groups checked significantly less under punishment, except for the most severe OCD and SCZ-OCS patients, who showed a positive relationship between punished checking and compulsive symptom severity as measured by YBOCS. Further analysis of possible factors affecting checking, such as intolerance of uncertainty, cognitive inflexibility, and working memory, was conducted in all four groups to better understand its psychological basis as well as its relationship, specifically in the two schizophrenia groups, with positive symptoms, anxiety, clozapine dose, and duration of treatment.

## Cognitive flexibility, working memory, and other clinical measures

As expected, SCZ-OCS and OCD patients showed enhanced depression, anxiety, intolerance of uncertainty, and OCS, and as previously reported (Biria et al., 2019), the SCZ-OCS group also exhibited increased positive and depressive symptoms (PANSS) compared with the SCZ-only group. In a recent mediation analysis, psychosis severity mediated checking behavior indirectly by inducing obsessions (Fernandez-Egea et al., 2024), and this could potentially explain the role of schizophrenia symptom severity in the development of checking behavior.

Cognitive flexibility, executive functioning, and spatial working memory are known deficits in schizophrenia patients (Bowie & Harvey, 2006; Goldberg & Green, 2002; Leeson et al., 2009; Pantelis et al., 1999) and we also replicated these findings by detecting worse performance on IED across all measures, digit span backward and the CANTAB spatial working memory test in both schizophrenia groups compared to OCD and healthy controls. The two schizophrenia groups were not different in cognitive performance across several measures of working memory, although the SCZ-OCS group were more impaired on the number of completed stages and PRE-EDS errors on the IED task compared to SCZ-only group. In a previous study by Patel et al. (2010), the deficit was apparent at the extra-dimensional shift stage of the IED task, perhaps because of differences in depressive symptom severity between the SCZ-OCS groups used in these studies.

## Checking under uncertainty

Our initial hypothesis of dysfunctional checking in both the SCZ-OCS and OCD groups was only confirmed in the former patient group. Checking under uncertainty was related to clozapine dose only in SCZ-OCS patients, despite no differences in clozapine dose between the two schizophrenia groups. While this implies a causal role for clozapine in inducing checking in SCZ-OCS patients, it is clear that clozapine dose alone is insufficient to cause this behavior and that other characteristics of SCZ-OCS patients, such as anxiety and executive dyscontrol (impaired working memory and cognitive inflexibility), enhance vulnerability to these effects of clozapine. It was also noticeable that checking in the SCZ-only group was functional, being related to their accuracy of perceptual decision-making. This is an important finding as it suggests that monitoring of performance was unimpaired in these patients, despite exhibiting other executive deficits. By contrast, such monitoring was clearly impaired in the SCZ-OCS group.

## Checking under punishment

The SCZ-OCS group had more severe symptoms compared to the SCZ-only group, as apparent from their higher anxiety, positive and depressive symptoms, and impaired performance at the earlier stage of the IED task. All these variables were also associated with punished checking, only in SCZ-OCS patients. Despite having similar working memory deficits on the CANTAB SWM task in both schizophrenia groups, again only SCZ-OCS patients exhibited a positive correlation between working memory impairment and punished checking. The lack of link between checking and working memory deficits in SCZ-only patients is consistent with a previous study, which measured checking in this clinical population using eye tracking (Jaafari et al., 2015). The SCZ-OCS patients showed a positive trend between checking under punishment and clozapine treatment duration. The latter finding is consistent with Fernandez-Egea et al. (2018) who showed that 25% of patients exhibit excessive checking behavior after 5 years of treatment, rising to over 50% after 10 years. Hence, it appears likely that excessive checking in SCZ-OCS patients is determined by clozapine acting in tandem with other factors, such as emotional state (anxiety) and executive dyscontrol.

## Possible neural and neurochemical substrates of dysfunctional checking associated with clozapine

Previous studies of excessive checking in OCD have implicated brain mechanisms in the anterior cingulate cortex (ACC), thalamus, and striatum (Biria et al., 2024; Mataix-Cols et al., 2004; Murayama et al., 2013), consistent with the role of the ACC in action monitoring and conflict resolution during decision-making under uncertainty (Alexander & Brown, 2011). White matter integrity in the cingulum, orbitofrontal cortex (OFC), and in fronto-striatal tracts is compromised more in SCZ-OCS in comparison with OCD or SCZ-only patients (Bıçakcı Ay et al., 2023). As clozapine antagonizes 5-HT2A and 5-HT2C receptors, it is relevant that OFC activation is enhanced in fMRI studies of schizophrenia patients treated with clozapine (Schirmbeck et al., 2013), opposite to therapeutic effects of SSRI treatment in OCD (Saxena et al., 1999). Using MRS, McQueen et al. (2021) reported that a 12-week of clozapine treatment was associated with a longitudinal reduction in Glx (glutamate + glutamine) in the caudate nucleus, which positively correlated with symptom improvement. However, the precise causal role of any of these changes in checking associated with clozapine treatment is not clear and requires further research.

Clinically, our results suggest that OCS in clozapine-treated patients emerge due to a combination of psychosis, depression and anxiety severity, clozapine load, and working memory dysfunction. The treatment might require an individualized assessment of these factors and intervention accordingly (e.g. improving depression or reducing clozapine dose when possible), albeit further studies are needed.

## Limitations

Although the IVT was able to characterize dysfunctional checking in SCZ-OCS patients, the lack of excessive checking behavior overall in both OCS groups was somewhat unexpected. A related study (Biria et al., 2024) used a modified version of the task that removed the time pressure component and led to excessive levels of checking in OCD patients and it would, therefore, be of interest to employ this in future studies of SCZ-OCS patients. In addition, we did not have the clozapine plasma levels available for all patients, and although we do show the relationship between clozapine plasma levels and clinical symptoms in a larger sample (n = 196) elsewhere (Fernandez-Egea et al., 2024), it would be important to study this relationship more precisely with checking on the IVT in the future.

Another limitation was the small sample size, which did not allow us to conduct a mediation analysis to investigate the relative causal importance of other factors such as working memory and anxiety, besides clozapine dose and treatment duration, which was partly due to our strict inclusion criteria in the schizophrenia groups. Additionally, the small sample size in the schizophrenia groups did not allow a mediation analysis to clarify the impact of demographic differences (e.g. age and sex) but also clinical and cognitive symptoms (e.g. positive symptoms and cognitive flexibility) on their IVT performance. We have, however, studied the sociodemographic and clinical aspects of clozapine treatment in relation to OCS separately with larger and more heterogeneous samples of clozapine-treated patients (Biria et al., 2019; Fernandez-Egea et al., 2018; Fernandez-Egea et al., 2024; Parkin et al., 2023).

Finally, since our study had a cross-sectional design, it also did not allow us to study the temporal trajectory of checking behavior development. Nevertheless, this study has provided us with an effective methodological platform for pursuing these additional questions.

## Conclusion

Dysfunctional checking was successfully measured in clozapine-treated schizophrenia patients with OCS and the factors contributing to this symptom were uncovered, including clozapine dose, state anxiety, and working memory deficits. The dysfunctional checking observed in schizophrenia patients with OCS was greater than in patients with schizophrenia only (without OCS) (matched for clozapine dose) and OCD. The checking in the OCD group was related to intolerance of uncertainty. The SCZ-OCS patients exhibited more severe PANSS symptomatology than schizophrenia patients without OCS (confirming previous findings) and clearly require special therapeutic approaches.

## Supporting information

Biria et al. supplementary material Biria et al. supplementary material

## Data Availability

The data used for this study can be found at: https://github.com/marbi515/SCZ_OCS_paper.

## Supplementary material

The supplementary material for this article can be found at http://doi.org/10.1017/S0033291724003350.
