# Supplementary material for: Clozapine-induced obsessive-compulsive symptoms in schizophrenia: Clinical and cognitive determinants of dysfunctional checking
Source: Psychol Med. 2025 Mar 18;55:e87. doi: 10.1017/S0033291724003350 (PMC12080643; doi:10.1017/S0033291724003350)
Supplement: Biria et al. supplementary material [file S0033291724003350sup001.pdf]

Supplement

Clozapine-induced obsessive-compulsive symptoms in schizophrenia: Clinical and cognitive determinants of dysfunctional checking

Marjan Biria[1,2,3], Paula Banca[1,2*], Isaac Jarratt Barnham[4,5*], Aleya A. Marzuki[1,6,7], Nuria Segarra[8], Engin Keser[1,9], Akeem Sule[10], Marija Farrugia[8], Qiang Luo[11], Naomi Fineberg[12], Emilio Fernandez-Egea[2,8,13], Trevor W. Robbins[1,2]

* : These authors contributed equally.

[1] Department of Psychology, University of Cambridge, Cambridge CB2 3EB, UK
[2] Behavioural and Clinical Neuroscience Institute, University of Cambridge, Cambridge CB2 3EB, UK
[3] Division of Psychiatry and Division of Psychology and Language Sciences, University College London, London, United Kingdom
[4] Cambridge Psychosis Centre, Cambridgeshire and Peterborough NHS Foundation Trust, Cambridge, UK
[5] Pembroke College, University of Oxford, St. Aldates, Oxford OX1 1DW
[6] Department of Psychiatry and Psychotherapy, Medical School and University Hospital, Eberhard Karls University of Tübingen, Tübingen, Germany
[7] German Center for Mental Health (DZPG), Tübingen, Germany
[8] Cambridgeshire and Peterborough NHS Foundation Trust
[9] Genetic and Developmental Psychiatry Centre, King's College London
[10] Department of Psychiatry, School of Clinical Medicine, University of Cambridge, Cambridge, UK
[11] Centre for Computational Psychiatry, Institute of Science and Technology for Brain-Inspired Intelligence, Fudan University, Shanghai 200433, China.

[12] Hertfordshire Partnership University NHS Foundation Trust, National Health Service, University of Hertfordshire, Hatfield, United Kingdom

[13] Cambridgeshire and Peterborough NHS Foundation Trust. Cambridge, UK

keywords: checking, spatial working memory, cognitive flexibility, schizophrenia, OCD.

Table.S1 Multiple comparison of means for demographics, cognitive and clinical data

Measures	Group I	Group J	Mean-difference(I-J)	Std. Error	p-adjusted	95% CI
Age (Years)	**HV**	**OCD**	4.585*	1.525	0.015	[0.65, 8.52]
	HV	SCZ-OCS	-3.729	1.707	0.13	[-8.13, 0.68]
	**HV**	**SCZ-only**	-6.367*	1.898	0.005	[-11.26, -1.47]
	**OCD**	**SCZ-OCS**	-8.314*	1.685	<.001	[-12.66, -3.97]
	**OCD**	**SCZ-only**	-10.952*	1.878	<.001	[-15.8, -6.11]
	SCZ-OCS	SCZ-only	-2.638	2.029	0.563	[-7.87, 2.6]
Verbal IQ (NART)	HV	OCD	-0.613	0.803	0.871	[-2.68, 1.46]
	HV	SCZ-OCS	-0.656	0.911	0.889	[-3.01, 1.7]
	HV	SCZ-only	2.578	1.006	0.053	[-0.02, 5.17]
	OCD	SCZ-OCS	-0.044	0.911	1	[-2.4, 2.31]
	**OCD**	**SCZ-only**	3.190*	1.006	0.009	[0.59, 5.79]
	**SCZ-OCS**	**SCZ-only**	3.234*	1.095	0.018	[0.41, 6.06]
Depression (MADRS)	**HV**	**OCD**	-7.121*	0.742	<.001	[-9.03, -5.21]
	**HV**	**SCZ-OCS**	-7.636*	0.831	<.001	[-9.78, -5.49]
	**HV**	**SCZ-only**	-3.017*	0.923	0.006	[-5.4, -0.63]
	OCD	SCZ-OCS	-0.515	0.82	0.923	[-2.63, 1.60]
	**OCD**	**SCZ-only**	4.104*	0.913	<.001	[1.75, 6.46]
	**SCZ-OCS**	**SCZ-only**	4.619*	0.987	<.001	[2.07, 7.17]
State anxiety (STAI-S)	**HV**	**OCD**	-13.131*	1.285	<.001	[-16.45, -9.81]
	**HV**	**SCZ-OCS**	-16.448*	1.428	<.001	[-20.13, -12.76]
	**HV**	**SCZ-only**	-11.467*	1.587	<.001	[-15.56, -7.37]
	OCD	SCZ-OCS	-3.316	1.419	0.091	[-6.98, 0.34]
	OCD	SCZ-only	1.665	1.579	0.717	[-2.41, 5.74]
	**SCZ-OCS**	**SCZ-only**	4.981*	1.697	0.018	[0.60, 9.36]
Trait anxiety (STAI-T)	**HV**	**OCD**	-22.280*	1.268	<.001	[-25.55, -19.01]
	**HV**	**SCZ-OCS**	-19.000*	1.409	<.001	[-22.63, -15.37]
	**HV**	**SCZ-only**	-10.267*	1.566	<.001	[-14.31, -6.23]
	OCD	SCZ-OCS	3.28	1.399	0.09	[-0.33, 6.89]
	**OCD**	**SCZ-only**	12.013*	1.557	<.001	[7.99, 16.03]
	**SCZ-OCS**	**SCZ-only**	8.733*	1.674	<.001	[4.41, 13.05]
IOU	**HV**	**OCD**	-27.333*	2.695	<.001	[-34.29, -20.38]
	**HV**	**SCZ-OCS**	-28.929*	3.017	<.001	[-36.71, -21.14]
	**HV**	**SCZ-only**	-12.767*	3.354	<.001	[-21.42, -4.11]
	OCD	SCZ-OCS	-1.595	2.978	0.95	[-9.28, 6.09]
	**OCD**	**SCZ-only**	14.567*	3.319	<.001	[6, 23.13]
	**SCZ-OCS**	**SCZ-only**	16.162*	3.585	<.001	[6.91, 25.41]
OCI (total score)	**HV**	**OCD**	-50.258*	3.004	<.001	[-58.01, -42.51]
	**HV**	**SCZ-OCS**	-44.776*	3.363	<.001	[-53.45, -36.10]
	**HV**	**SCZ-only**	-16.233*	3.737	<.001	[-25.88, -6.59]
	OCD	SCZ-OCS	5.482	3.319	0.351	[-3.08, 14.05]
	**OCD**	**SCZ-only**	34.025*	3.698	<.001	[24.48, 43.57]
	**SCZ-OCS**	**SCZ-only**	28.543*	3.995	<.001	[18.23, 38.85]
Digit span forward	**HV**	**OCD**	-1.277*	0.328	<.001	[-2.12, -0.43]
	HV	SCZ-OCS	0.162	0.364	0.971	[-0.78, 1.1]
	HV	SCZ-only	-0.333	0.405	0.843	[-1.38, 0.71]
	**OCD**	**SCZ-OCS**	1.439*	0.362	<.001	[0.51, 2.37]
	OCD	SCZ-only	0.944	0.402	0.09	[-0.09, 1.98]
	SCZ-OCS	SCZ-only	-0.495	0.433	0.662	[-1.61, 0.62]
Digit span backwards	**HV**	**OCD**	-.962*	0.29	0.006	[-1.71, -0.21]
	**HV**	**SCZ-OCS**	1.119*	0.323	0.003	[0.29, 1.95]
	**HV**	**SCZ-only**	1.100*	0.359	0.012	[0.17, 2.03]
	**OCD**	**SCZ-OCS**	2.081*	0.32	<.001	[1.25, 2.91]
	**OCD**	**SCZ-only**	2.062*	0.357	<.001	[1.14, 2.98]
	SCZ-OCS	SCZ-only	-0.019	0.383	1	[-1.01, 0.97]
IED completed stages	HV	OCD	0.075	0.204	0.983	[-0.45, 0.60]
	**HV**	**SCZ-OCS**	1.560*	0.226	<.001	[0.98, 2.14]
	HV	SCZ-only	0.389	0.251	0.41	[-0.26, 1.04]
	**OCD**	**SCZ-OCS**	1.485*	0.223	<.001	[0.91, 2.06]
	OCD	SCZ-only	0.314	0.248	0.586	[-0.33, 0.95]
	**SCZ-OCS**	**SCZ-only**	-1.171*	0.267	<.001	[-1.86, -0.48]
IED PRE-EDS errors	HV	OCD	1.623	0.779	0.16	[-0.39, 3.63]
	**HV**	**SCZ-OCS**	-6.964*	0.864	<.001	[-9.19, -4.73]
	HV	SCZ-only	-1.678	0.959	0.299	[-4.15, 0.8]
	**OCD**	**SCZ-OCS**	-8.587*	0.852	<.001	[-10.79, -6.39]
	**OCD**	**SCZ-only**	-3.301*	0.948	0.003	[-5.75, -0.85]
	**SCZ-OCS**	**SCZ-only**	5.286*	1.019	<.001	[2.66, 7.92]

Measures	Group I	Group J	Mean-difference(I-J)	Std. Error	p-adjusted	95% CI
IED EDS errors	HV	OCD	-2.855	1.19	0.079	[-5.93, 0.21]
	**HV**	**SCZ-OCS**	-9.901*	1.319	<.001	[-13.31, -6.5]
	**HV**	**SCZ-only**	-9.092*	1.465	<.001	[-12.87, -5.31]
	**OCD**	**SCZ-OCS**	-7.046*	1.301	<.001	[-10.4, -3.69]
	**OCD**	**SCZ-only**	-6.237*	1.448	<.001	[-9.97, -2.5]
	SCZ-OCS	SCZ-only	0.81	1.557	0.954	[-3.21, 4.83]
SWMBE	HV	OCD	-2.116	1.182	0.28	[-5.17, 0.94]
	**HV**	**SCZ-OCS**	-7.917*	1.309	<.001	[-11.3, -4.54]
	**HV**	**SCZ-only**	-7.202*	1.451	<.001	[-10.95, -3.46]
	**OCD**	**SCZ-OCS**	-5.800*	1.282	<.001	[-9.11, -2.49]
	**OCD**	**SCZ-only**	-5.086*	1.427	0.002	[-8.77, -1.4]
	SCZ-OCS	SCZ-only	0.714	1.533	0.966	[-3.24, 4.67]

Acronyms: HV = healthy volunteers, OCD = obsessive compulsive disorder, SCZ-OCS = schizophrenia with OCS, SCZ-only = schizophrenia without OCS, *MADRS* Montgomery-Asberg Depression Rating Scale, *STAI-S* State Trait Anxiety Inventory-State, *STAI-T* State Trait Anxiety Inventory-Trait, *IOU* Intolerance Of Uncertainty, *OCI* Obsessive Compulsive Inventory, *IED* Intra-Extra Dimensional Set Shift, *ED* extradimensional shift, *SWMTE* Spatial Working Memory Total Errors, *SWMBE* SWM Between Errors (the number of times the subject incorrectly revisits a box in which a token has previously been found). The asterisks and bold letters mark the significant comparisons.

Table.S2 Descriptive statistics of the Image Verification Task performance

Measure	Group	Block	Mean	SD	95% CI
Confidence (mean)	HV	1	3.36	0.31	[3.24, 3.48]
	OCD	1	3.23	0.35	[3.10, 3.35]
	SCZ-OCS	1	3.22	0.47	[3.01, 3.43]
	SCZ-only	1	3.08	0.36	[2.88, 3.28]
	HV	2	3.10	0.31	[2.98, 3.22]
	OCD	2	2.93	0.50	[2.74, 3.11]
	SCZ-OCS	2	2.96	0.60	[2.69, 3.24]
	SCZ-only	2	2.94	0.50	[2.66, 3.22]
Accuracy (%)	HV	1	78.70	4.81	[76.90, 80.50]
	OCD	1	78.47	7.36	[75.81, 81.12]
	SCZ-OCS	1	69.86	7.74	[66.33, 73.38]
	SCZ-only	1	71.13	8.64	[66.35, 75.92]
	HV	2	77.13	7.72	[74.25, 80.02]
	OCD	2	76.09	8.82	[72.91, 79.27]
	SCZ-OCS	2	69.05	9.32	[64.81, 73.29]
	SCZ-only	2	69.60	7.14	[65.65, 73.55]
Checking rate (sum)	HV	1	19.90	13.80	[14.75, 25.05]
	OCD	1	21.03	15.66	[15.38, 26.68]
	SCZ-OCS	1	12.00	14.58	[5.36, 18.64]
	SCZ-only	1	11.33	10.99	[5.25, 17.42]
	HV	2	5.03	8.26	[1.95, 8.12]
	OCD	2	5.31	7.19	[2.72, 7.91]
	SCZ-OCS	2	6.10	7.89	[2.50, 9.69]
	SCZ-only	2	5.80	10.39	[0.04, 11.56]

Acronyms: *HV*, healthy volunteers, OCD, obsessive compulsive disorder, SCZ-OCS, schizophrenia with obsessive compulsive symptoms, SCZ-only, schizophrenia without obsessive compulsive symptoms, SD, standard deviation, *CI*, confidence interval of the mean.

Table.S3 Multiple comparison of means for Image Verification Task performance

Measures	Group I	Group J	Mean-difference(I-J)	Std. Error	p-adjusted	95% CI
Confidence-block 1	HV	OCD	0.14	0.09	0.45	[-0.10, 0.38]
	HV	SCZ-OCS	0.14	0.10	0.54	[-0.13, 0.41]
	HV	SCZ-only	0.28	0.12	0.08	[-0.02, 0.59]
	OCD	SCZ-OCS	0.00	0.10	1.00	[-0.26, 0.27]
	OCD	SCZ-only	0.14	0.11	0.59	[-0.15, 0.44]
	SCZ-OCS	SCZ-only	0.14	0.12	0.66	[-0.18, 0.47]
Accuracy-block 1	HV	OCD	0.23	1.78	1.00	[-4.42, 4.88]
	**HV**	**SCZ-OCS**	8.843*	1.99	<.001	[3.64, 14.05]
	**HV**	**SCZ-only**	7.567*	2.21	0.01	[1.78, 13.35]
	**OCD**	**SCZ-OCS**	8.612*	1.96	<.001	[3.48, 13.75]
	**OCD**	**SCZ-only**	7.335*	2.19	0.01	[1.61, 13.06]
	SCZ-OCS	SCZ-only	-1.28	2.36	0.95	[-7.46, 4.91]
Checking-block 1	HV	OCD	-1.13	3.62	0.99	[-10.60, 8.33]
	HV	SCZ-OCS	7.90	4.05	0.22	[-2.70, 18.50]
	HV	SCZ-only	8.57	4.50	0.23	[-3.21, 20.35]
	OCD	SCZ-OCS	9.03	4.00	0.12	[-1.43, 19.49]
	OCD	SCZ-only	9.70	4.46	0.14	[-1.96, 21.35]
	SCZ-OCS	SCZ-only	0.67	4.81	1.00	[-11.93, 13.26]
Confidence-block 2	HV	OCD	0.18	0.12	0.47	[-0.14, 0.49]
	HV	SCZ-OCS	0.14	0.14	0.74	[-0.21, 0.49]
	HV	SCZ-only	0.16	0.15	0.71	[-0.23, 0.56]
	OCD	SCZ-OCS	-0.04	0.13	0.99	[-0.38, 0.31]
	OCD	SCZ-only	-0.01	0.15	1.00	[-0.40, 0.38]
	SCZ-OCS	SCZ-only	0.02	0.16	1.00	[-0.39, 0.44]
Accuracy-block 2	HV	OCD	1.04	2.13	0.96	[-4.53, 6.60]
	**HV**	**SCZ-OCS**	8.086*	2.38	0.01	[1.86, 14.32]
	**HV**	**SCZ-only**	7.533*	2.65	0.03	[0.61, 14.46]
	**OCD**	**SCZ-OCS**	7.046*	2.35	0.02	[0.90, 13.20]
	OCD	SCZ-only	6.49	2.62	0.07	[-0.36, 13.35]
	SCZ-OCS	SCZ-only	-0.55	2.83	1.00	[-7.96, 6.85]
Checking-block 2	HV	OCD	-0.28	2.09	1.00	[-5.74, 5.18]
	HV	SCZ-OCS	-1.06	2.34	0.97	[-7.17, 5.05]
	HV	SCZ-only	-0.77	2.60	0.99	[-7.56, 6.06]
	OCD	SCZ-OCS	-0.78	2.31	0.99	[-6.81, 5.25]
	OCD	SCZ-only	-0.49	2.57	1.00	[-7.21, 6.23]
	SCZ-OCS	SCZ-only	0.30	2.78	1.00	[-6.97, 7.56]

Acronyms: HV = healthy volunteers, OCD = obsessive compulsive disorder, SCZ-OCS = schizophrenia with OCS, SCZ-only = schizophrenia without OCS, SD, standard deviation, *CI*, confidence interval of the mean. The asterisks mark the significant comparisons.

Contrast analysis and IVT group differences

We analysed 3 contrasts (C1, C2, C3) comparing the checking, confidence rating and accuracy of answers between C1) HV vs patients, C2) OCD vs schizophrenia, and C3) SCZ-OCS vs SCZ-only groups for block 1 (high uncertainty, no feedback) and block 2 (punished checking, feedback). The orange lines specify significant group differences, whereas, the blue lines show the significant differences in blocks. For C1: (**Fig.S1.A**) 1) *checking*: there was a main effect of block ($F(1, 96)= 38, 32$, $p < 0.001$, ges = 0.04) and no group or interaction effects. This means that both groups checked significantly less after punishment was introduced in second block. 2) *confidence*: there was a main effect of group ($F(1, 96)= 4.01$, $p = 0.04$, ges = 0.03) and block ($F(1, 96)= 65.92$, $p < 0.001$, ges = 0.05). This means that healthy subjects were more confident than patients overall, and both groups were more confident in first block without feedback compared to second block with punishment. 3) *accuracy*: there was a main effect of group ($F(1, 96)= 10.26$, $p = 0.002$, ges = 0.07), and no block or interaction effects. This means that healthy participants had an overall better performance than patients with no difference between blocks. For C2 (**Fig. S1.B**): 1) *checking*: there were main effects of group ($F(1, 66)= 7.49$, $p = 0.007$, ges = 0.09) and block ($F(1, 66)= 19.61$, $p < 0.001$, ges = 0.04) but no interaction effects. Patients with OCD checked significantly more than schizophrenia groups, and there was a reduction in checking from block 1 to block 2. 2) *confidence:* there was a main effect of block ($F(1, 66)= 37.63$, $p < 0.001$, ges = 0.04) but no group or interactions effects. This means that patients with OCD and schizophrenia were more confident in block 1 than block 2. 3) *accuracy*: there was a main effect of group ($F(1, 66)= 29.13$, $p < 0.001$, ges = 0.23) but no block or interaction effects. OCD group was more accurate than the schizophrenia groups in both blocks . Lastly, for C3 (**Fig. S1.C**): 1) *checking*: there was only a main effect of block ($F(1, 96)= 4.45$, $p = 0.04$, ges = 0.02) with both groups checking less in second block where checking was punished, 2) *confidence*: again, there was only a main effect of block ($F(1, 96)= 12.18$, $p = 0.001$, ges = 0.02) with both groups being more confident in block 1 compared to the second block, and 3) accuracy: no main effects.

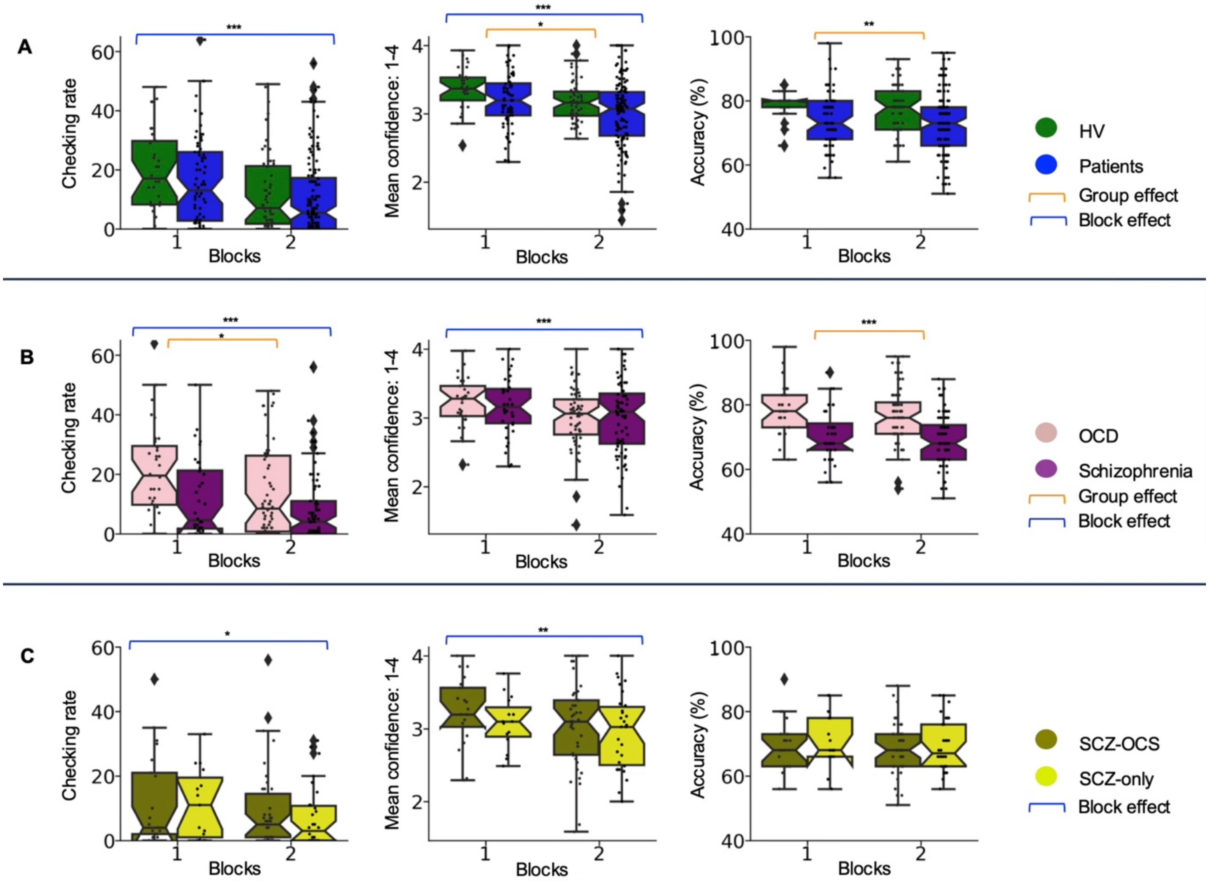

Fig.S1 Independent contrast analyses for performance on the Image verification task. The columns from left to right represent checking rate, mean confidence ratings (ranging from 1 to 4), and accuracy of choices (%). The boxplots show comparisons from (A) Contrast 1: between healthy volunteers (HVs; N = 30) in green and patients (N = 69) in blue, (B) Contrast 2: between OCD (N = 31) in pink and the two schizophrenia groups (N = 38) in purple, and (C) Contrast 3: between SCZ-OCS patients (N = 23) in olive green and SCZ-only group (N = 23) in yellow. One SCZ-only patient was excluded from the accuracy analysis and the plot as they had an accuracy lower than 2SD from the mean. The black dots represent the individual data points; the boxes start from the first to the third quartile, with a horizontal line and a notch through the median. The whiskers go from each quartile to the minimum and maximum. The notch provides an estimated 95% confidence interval for the median. When the notches of two boxes don't intersect, it implies a significant difference in the medians. Data points lying beyond the whiskers are identified as outliers. Blue lines above the plots specify differences in main effect of blocks, whereas the orange lines mark the differences in main effects for group. Acronyms: HV = healthy volunteers, OCD = obsessive compulsive disorder, SCZ-OCS = schizophrenia with OCS, SCZ-only = schizophrenia without OCS, * $p < .05$, ** $p < .01$, *** $p < 0.001$.

Medication status

SCZ-OCS group: 13 were treated solely with clozapine, the rest were on a combination of medications. One was on clozapine and SSRI, 8 on clozapine, SSRI and aripiprazole, and 1 on clozapine, SSRI, and lithium.

SCZ-only group: 3 were treated with clozapine alone, 4 were on clozapine and SSRIs, 6 on clozapine, SSRIs and aripiprazole, 1 on clozapine and aripiprazole, and 1 was on clozapine, sulpiride and clomipramine.

OCD group: all but 9 patients were medicated. Out of the 22 medicated patients, 19 were prescribed only with SSRIs, 3 were treated with adjunct antipsychotics, and 1 was on clomipramine and propranolol.

Missing data

The following data were missing: 1 CANTAB Intra-Extra Dimensional Set Shift (IED), and 2 CANTAB Spatial Working Memory (SWM) in healthy control group, 2 Verbal IQ (NART), 1 digit span and STAI (State/Trait Anxiety Questionnaire), 1 IED and SWM in OCD patients and 1 Verbal IQ for schizophrenia patient without OCS, and 2 Verbal IQ's from schizophrenia patients with OCS. The latter 2 missing Verbal IQ's in schizophrenia patients was due having dyslexia diagnoses. Education years were only compared between OCD and healthy control group as this information was not available for most schizophrenia patients.

R Code and output for the contrast analyses

```
library(dplyr)
library(tidyr)
library(broom)
library(ez)

df$Accuracy_percentage <- (df$Accuracy_.)*100
df$block <- as.factor(df$block)
df <- df %>%
  mutate(block = recode(block, `3` = "2"))
df <- subset(df, block %in% c(1,2))
df_B1 <- subset(df, block %in% c(1))
df_B3 <- subset(df, block %in% c(2))

df_c1 <- df %>%
  filter(HV_patients != "exclude")
df_c2 <- df %>%
  filter(OCD_Schizophrenia != "exclude")
df_c3 <- df %>%
  filter(Sch_OCD_Sch_No_OCD != "exclude")

# repeated ANOVA for contrast 1: HV vs patients
result_ch <- ezANOVA(
  data = df_c1,
  dv = .(checking_rate),
  wid = .(ID),
  within = .(block),
  between = .(HV_patients)
); print(result_ch)
```

ANOVA FOR CHECKING:

	Effect	DFn	DFd	F	p	p<.05	ges
2	HV_patients	1	96	1.106734	2.954338e-01		0.010016103
**3**	**block**	**1**	**96**	**38.319726**	**1.478631e-08**	*****	**0.046580423**
4	HV_patients:block	1	96	1.103568	2.961218e-01		0.001405029

```
result_conf <- ezANOVA(
  data = df_c1,
  dv = .(mean_confidence_all),
  wid = .(ID),
  within = .(block),
  between = .(HV_patients)
);print(result_conf)
```

ANOVA FOR CONFIDENCE:

	Effect	DFn	DFd	F	p	p<.05	ges
**2**	**HV_patients**	**1**	**96**	**4.010000**	**4.805178e-02**	*****	**3.676262e-02**
**3**	**block**	**1**	**96**	**65.92682**	**1.585933e-12**	*****	**5.595444e-02**
4	HV_patients:block	1	96	0.008037	9.287497e-01		7.226147e-06

```
result_acc<- ezANOVA(
  data = df_c1,
  dv = .(Accuracy_percentage),
  wid = .(ID),
  within = .(block),
  between = .(HV_patients)
);print(result_acc)
```

ANOVA FOR ACCURACY:

	Effect	DFn	DFd	F	p	p<.05	ges
**2**	**HV_patients**	**1**	**96**	**10.263265**	**0.001842041**	*****	**0.075671354**
3	block	1	96	3.587818	0.061214974		0.008678428
4	HV_patients:block	1	96	0.143236	0.705920510		0.000349379

```
# repeated ANOVA for contrast 2: OCD vs schizophrenia
result_ch <- ezANOVA(
  data = df_c2,
  dv = .(checking_rate),
  wid = .(ID),
  within = .(block),
  between = .(OCD_Schizophrenia)
);print(result_ch)
```

ANOVA FOR CHECKING:

	Effect	DFn	DFd	F	p	p<.05	ges
**2**	**OCD_Schizophrenia**	**1**	**66**	**7.492958**	**7.952146e-03**	*****	**0.08925724**
**3**	**block**	**1**	**66**	**19.617271**	**3.643656e-05**	*****	**0.03905782**
4	OCD_Schizophrenia:block	1	66	1.968442	1.653004e-01		0.00406188

```
result_conf <- ezANOVA(
  data = df_c2,
  dv = .(mean_confidence_all),
  wid = .(ID),
  within = .(block),
  between = .(OCD_Schizophrenia)
);print(result_conf)
```

ANOVA FOR CONFIDENCE:

	Effect	DFn	DFd	F	p	p<.05	ges
2	OCD_Schizophrenia	1	66	0.065151	7.993247e-01		0.0009013836
**3**	**block**	**1**	**66**	**37.629126**	**5.419698e-08**	*****	**0.0467709563**
4	OCD_Schizophrenia:block	1	66	1.293862	2.594500e-01		0.0016842671

```
result_acc<- ezANOVA(
  data = df_c2,
  dv = .(Accuracy_percentage),
  wid = .(ID),
  within = .(block),
  between = .(OCD_Schizophrenia)
);print(result_acc)
```

ANOVA FOR ACCURACY:

	Effect	DFn	DFd	F	p	p<.05	ges
**2**	**OCD_Schizophrenia**	**1**	**66.**	**29.131883**	**9.868167e-07**	*****	**0.2340569075**
3	block	1	66	2.549233	1.151252e-01		0.0117448837
4	OCD_Schizophrenia:block	1	66	0.076843	7.824874e-01		0.0003581158

```
# repeated ANOVA for contrast 3: schizo_OCS vs SCZ-only
result_ch <- ezANOVA(
  data = df_c3,
  dv = .(checking_rate),
  wid = .(ID),
  within = .(block),
  between = .(Sch_OCD_Sch_No_OCD)
```

```
);print(result_ch)
```

ANOVA FOR CHECKING:

	Effect	DFn	DFd	F	p	p<.05	ges
2	Sch_OCD_Sch_No_OCD	1	34	0.1473758	0.70344617		0.0035347264
**3**	**block**	**1**	**34**	**4.4593919**	**0.04213651**	*****	**0.0232688496**
4	Sch_OCD_Sch_No_OCD:block	1	34	0.1736183.	0.67953492		0.0009266527

```
result_conf <- ezANOVA(
  data = df_c3,
  dv = .(mean_confidence_all),
  wid = .(ID),
  within = .(block),
  between = .(Sch_OCD_Sch_No_OCD)
);print(result_conf)
```

ANOVA FOR CONFIDENCE:

	Effect	DFn	DFd	F	p	p<.05	ges
2	Sch_OCD_Sch_No_OCD	1	34	0.5275630	0.472604289		0.0140784910
**3**	**block**	**1**	**34**	**12.1872125**	**0.001353928**	*****	**0.0277826988**
4	Sch_OCD_Sch_No_OCD:block	1	34	0.3786697	0.542413433		0.0008871188

```
result_acc<- ezANOVA(
  data = df_c3,
  dv = .(Accuracy_percentage),
  wid = .(ID),
  within = .(block),
  between = .(Sch_OCD_Sch_No_OCD)
);print(result_acc)
```

ANOVA FOR ACCURACY:

	Effect	DFn	DFd	F	p	p<.05	ges
2	Sch_OCD_Sch_No_OCD	1	34	0.003526	0.9529908		6.062334e-05
3	block	1	34	1.322544.	0.2581609		1.590701e-02
4	Sch_OCD_Sch_No_OCD:block	1	34	0.468658	0.4982453		5.695321e-03
